# The efficacy and safety of qiming granule in dry eye disease: Protocol for systematic review and meta-analysis

**DOI:** 10.1097/MD.0000000000017121

**Published:** 2019-09-27

**Authors:** Maoyi Yang, Zhipeng Hu, Rensong Yue, Liangjun Yang, Boxun Zhang, Yuan Chen

**Affiliations:** a Hospital of Chengdu University of Traditional Chinese Medicine; b Pi Wei Institute, Guangzhou University of Chinese Medicine, Guangzhou, China.

**Keywords:** dry eye disease, protocol, qiming granule, Schirmer test, tear break-up time

## Abstract

**Background:**

Dry eye disease is a common eye disease mainly manifests with eye fatigue, foreign body sensation, dry and astringent eyes and other symptoms. Growing evidence shows that qiming granule may have beneficial effects on the clinical treatment of dry eye disease. However, no systematic review and meta-analysis collate and assess these clinical evidences. The purpose of this study protocol is to provide a comprehensive and reliable evaluation of the clinical evidence of qiming granule in the treatment of DED.

**Methods and analysis:**

Three English database and 4 Chinese databases other sources will be searched. Two methodological trained researchers will read the title, abstract and full texts and independently select the qualified literature according to inclusion and exclusion criteria. After assessment of the risk of bias and data extraction, we will conduct meta-analyses for outcomes including central macular thickness, optimum corrected vision, overall effect rates and adverse effects. The heterogeneity of data will be investigated by Cochrane X^2^ and *I*^*2*^ tests. We build 3 hypotheses for subgroup analysis according to the guidance for a credible subgroup effect: Disease status at baseline, duration of intervention, type of concomitant medication. Sensitivity analysis will be conducted to evaluate the stability of the results. Then publication bias assessment will be conducted by funnel plot analysis and Egger test. Finally, we will use the Grading of Recommendations Assessment, Development and Evaluate system to evaluate the quality of evidence.

**Results:**

The results of our research will be published in a peer-reviewed journal.

**Conclusion:**

Our study is the first meta-analysis to evaluate the clinical efficacy and safety of qiming granule in the treatment of DED. It will provide more options for clinical treatment of the disease.

**PROSPERO registration number:**

CRD42018109183.

## 1 Introduction

Dry eye disease (DED) is one of the most common ocular surface diseases in clinical practice which is caused by multifactor and characterized by eye discomfort and visual dysfunction. Its main manifestations include eye fatigue, foreign body sensation, dry and astringent eyes and other symptoms. The main pathophysiological mechanisms of DED are tear film instability, increased tear osmotic pressure, ocular surface inflammation and injury, and neurosensory abnormalities. DED is one of the most common eye diseases in clinical practice and it has an important impact on the quality of life of patients. In the United States, more than 16 million adults (6.8% of the total) are diagnosed with DED.^[1]^ In China, the incidence of DED is about 21% to 30%.^[2]^ The incidence of DED around the world is about 5% to 34%, and it increases with age.^[3–6]^ Despite the prevalence of DED, there are still challenges in the treatment of the disease.

Major treatments for DED include artificial tear replacement and environmental coping strategies. Artificial tear replacement is the first-line treatment for patients, which is effective in relieving symptoms.^[7,8]^ However, this treatment can only alleviate the symptoms, but cannot fundamentally improve the condition. Environmental coping strategies focus mainly on external causes that may lead to DED. But the disadvantage of this treatment strategy is that it can only delay the onset of diseases to a certain extent, but cannot play a therapeutic role in the management of diseases. Other treatments include topical use of cyclosporine, ritasteride and other medicines. However, there are insufficient evidence to prove the efficacy of these treatments, and these treatments have many side effects.^[9,10]^ Thus, it is still necessary to explore new treatment.

Qiming granule (QG) is a famous Chinese patent medicine, which consists of Huangqi (*Astragalus membranaceus*), Gegen (*Pueraria root*), Dihuang (*Rehmannia glutinosa*), Gouqi (*wolfberry*), Jueming Zi (*cassia seed*), Chongwei Zi (*motherwort fruit*), Puhung (*cattail*) and Shuizhi (*leech*). In China, QG is widely used in the treatment of diabetic retinopathy.^[11]^ In the guidelines for the prevention and treatment of diabetes promulgated in 2017 in China, it was mentioned as a candidate drug for diabetic retinopathy.^[12]^ In recent years, a series of clinical studies have suggested that QG has therapeutic effects on DED.^[13–16]^ However, there is still a lack of systematic review to comprehensively analyze the clinical evidence of it in the treatment of DED. Therefore, in this study, we will systematically collect clinical evidence of QG in the treatment of DED, and conduct a meta-analysis to evaluate the efficacy and safety of it on DED. Our results will provide evidence-based guidance for QG in the treatment of DED.

## 2 Methods and analysis

### 2.1 Study registration

We registered our study in the website of https://www.crd.york.ac.uk/ according to the Preferred Reporting Items for Systematic Reviews and Meta-analysis (PRISMA) of Observation Studies in Epidemiology recommendations for study reporting. This systematic review and meta-analysis protocol is reported according to the Preferred Reporting Items for Systematic Reviews and Meta-analysis Protocols (PRISMA-P) checklist.^[17]^ The PROSPERO registration number is CRD42018109183.

### 2.2 Inclusion and exclusion criteria

#### 2.2.1 Study design

The design types of studies included in our research will be limited to randomized controlled trial (RCT). Those studies that were not RCT including observational study and non-randomized controlled trials will not be included in the study. Real world study will not be included in our research either.

#### 2.2.2 Participants

All patients who were clinically diagnosed as DED will be included in our research, there will be no limitation about age, region, gender, disease severity, and other factors.

#### 2.2.3 Interventions

Those studies which set QG alone as an experimental treatment will be included in our research. Studies that combine QG with other treatment as experimental therapies will not be included. Control therapy can be any type of treatment.

#### 2.2.4 Outcomes

Any outcome that is related to the condition can be included. The primary outcome is total effective rate, this is judged based on the alleviation of symptoms and the improvement of laboratory examinations together. Secondary outcomes include tear break-up time, Schirmer test, Clinical symptoms and so on. Adverse reactions after taking the medicine will also be included in the study.

### 2.3 Study search

Three English database including PubMed, Embase, Cochrane Library Central Register of Controlled Trials and four Chinese databases including China National Knowledge Infrastructure (CNKI) database, Wanfang Data Knowledge Service Platform, the VIP information resource integration service platform (cqvip), China Biology Medicine Disc (Sino Med) with a language limitation of English and Chinese will be searched. In addition, we will also search Google scholar, Baidu Scholar to find out unpublished researches or other related literature. And above all, the Chinese Clinical Trial Registry (ChiCTR) and ClinicalTrials.gov will also be searched. A manual search will be conducted at the library of Chengdu University of Traditional Chinese Medicine.

A search strategy that combines MeSH terms and free words will be adopted by us. The search terms used will be as follows: “qiming granule”, “qiming”, “qiming keli”, “Dry Eye Syndrome”, “Syndrome, Dry Eye”, “Syndromes, Dry Eye”, “dry eye disease”, “dry eye”. Two authors (Zhipeng Hu and Maoyi Yang) will search and screen all the citations independently. The search process of the Cochrane library is presented in Table 1.

**Table 1:**
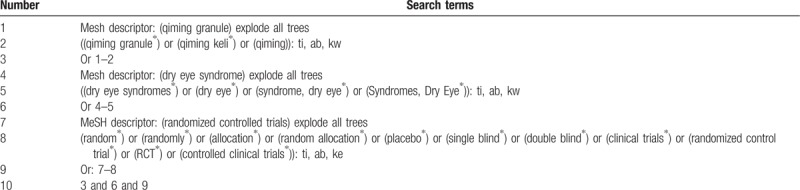
Example of Cochrane search strategy.

### 2.4 Study selection

**Figure 1:**
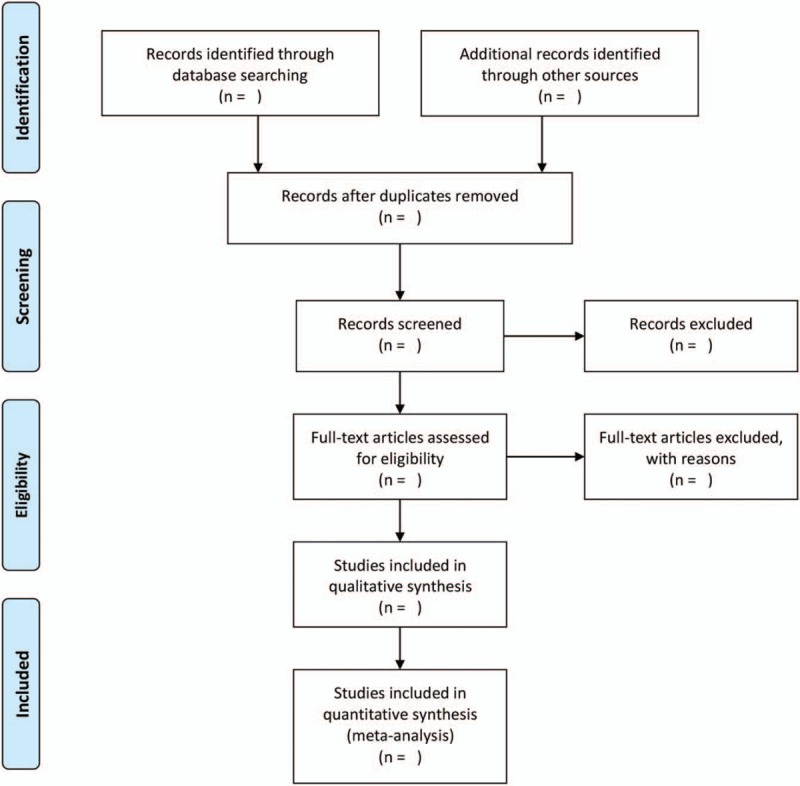
Flow chart of study selection.

We will manage the electronic citations we download from the above databases in Endnote X8 for windows (Thomson Reuters, USA). And then two methodological trained researchers will read the titles and abstracts of citations and screen the citations according to the inclusion and exclusion criteria. Those studies that meet the criteria will be further determined for inclusion by reading the full text. A final decision will be made through consensus when there were discrepancies. A flow chart of study selection will be drawn to show the whole process of study selection (Fig. 1).

### 2.5 Data extraction

We will extract the data of those qualified articles into Microsoft Excel according to a pre-set form. For each study, the following data will be extracted: The first authors of the article, year of publication, interventions in experimental group, interventions in control group, time of treatment, course of disease, number of patients in each group, ages and sex of patients, outcomes and safety data. If there is not enough data in a study, we will contact the corresponding author for more detailed data. If the methodological details are not told in papers, we will contact for more explanation.

### 2.6 Risk of bias assessment

The risk of bias of included articles will be assessed by two reviewers via the Cochrane collaboration's tool. This is an established and reliable tool for assessing the risk of bias in studies. In this tool, the risk of bias of a trial is assessed through these aspects: random sequence generation (selection bias), allocation concealment (selection bias), blinding of participants and personnel (performance bias), blinding of outcome assessment (detection bias), incomplete outcome data (attrition bias), selective reporting (reporting bias), other bias. Each item is classified as “Low risk”, “High risk” or “Unclear risk”.^[18]^ Two reviewers will conduct the risk of bias assessment independently and any disagreements will be solved by a discussion of all reviewers.

### 2.7 Data analysis

Endnote X8 for windows (Thomson Reuters, USA) will be used to manage our citations, and Review Manager Version 5.3 and stata 14.0 software will be used to create forest plots and conduct subgroup analysis and sensitivity analysis. For the binary variable, the effect size will be represented as risk ratio (RR) and 95% confidence interval (CI) and a Mantel-Haenszel (M-H) method will be used to calculate them. For continuous variable, the effect size will be represented as mean difference (MD) and 95% CI. If one studies report its standard error (SEM) other than Standard Deviation (SD), we will convert SEM into SD. The heterogeneity of data will be investigated by Cochrane X^2^ and *I*^*2*^ tests.^[19]^ The statistical heterogeneity is considered substantial when *P* < .05 and *I*^*2*^ > 50%. If *P* > .05 and *I*^*2*^ < 50%, then the studies included is homogeneous and the differences between them can be ignored. If there is significant heterogeneity, the random effect model will used to pool data, and if there is no significant heterogeneity, then the fixed effect model will be used. If quantitative synthesis is not appropriate due to substantial heterogeneity, then the results will be presented with tables and figures.

### 2.8 Investigation of heterogeneity

If there is substantial heterogeneity between studies, then we will conduct subgroup analysis and meta-regression to explore the heterogeneity. We build three hypotheses for subgroup analysis: Disease status at baseline, duration of intervention, type of concomitant medication.^[20]^ We will conduct our subgroup analysis according to these subgroup hypotheses. Then we will evaluate the credibility of our subgroup analysis according to the guidance for credible subgroup analysis.^[21]^ If there are enough studies, then meta regression will be conducted to further explore the sources of heterogeneity.

### 2.9 Sensitivity analysis

Then we will conduct a sensitivity analysis to investigate the stability of the results. We will exclude each study that is included in the analysis one by one, and then re-analyze and pooled the data and compare the difference between the re-obtained effects and the original effects. In this way, we will be able to assess the impact of individual studies on the overall results and whether the results are robust.

### 2.10 Publication bias assessment

If there are more than 10 studies included, a funnel plot analysis will be drawn to assess the publication bias and Egger test will be conducted for statistical investigation.^[22,23]^ The publication bias is considered to exist if *P* < .05.

### 2.11 Summary of finding tables

At last, the summary of finding tables for each outcome will be generated by Grading of Recommendations Assessment, Development and Evaluate system (GRADE). This is a widely used tool in evaluating the quality of assessment.^[24]^ In this table, the evidence will be shown from 5 domains: certainty assessment, number of patients, effect, certainty and importance. In the GRADE system, the quality of evidence can be defined as “high”, “moderate”, “low”, and “very low”.

### 2.12 Patient and public involvement

Patient and public were not involved in this study.

### 2.13 Ethics and dissemination

Ethical approval is not needed for this meta-analysis. Our study comprehensively evaluates the existing research evidence of qiming granule and is bound to provide evidence-based medical support for clinical workers. The results of our research will be published at a peer reviewed journal.

## 3 Discussion

DED is one of the most common eye diseases in clinic, and it is also an important disease affecting the quality of life of patients. At present, we can only take limited measures to control the symptoms of DED. In recent years, many clinical studies have suggested that QG may have a good effect on the disease. However, there is no meta-analysis to systematically collate and assess these clinical evidences. In order to provide more evidence-based medical support for clinical treatment, we conducted this systematic review and meta-analysis.

In this study, we will systematically collect clinical evidence and pool the results to investigate the efficacy of QG in the treatment of DED and evaluate the quality of the evidence. We will use a bias risk assessment tool to assess the bias risk of the included studies, and then use GRADE grading tool to classify the quality of evidence. In this way, our research will be able to better serve clinical decision-making. We will use subgroup analysis and meta-regression to explore the sources of heterogeneity between the included studies. In order to avoid meaningless results from post-analysis, we presupposed some features for subgroup analysis before the study began. Our subgroup analysis will be based on these subgroup assumptions. In addition, we will evaluate the reliability of subgroup analysis according to the criteria of reliability of subgroup analysis. Through these methods, we will be able to ensure that the subgroup analysis results of our study are credible.

Our study is the first meta-analysis to evaluate the clinical efficacy and safety of QG in the treatment of DED. It will provide more options for clinical treatment of the disease.

## Author contributions

**Conceptualization:** Maoyi Yang, Zhipeng Hu, Rensong Yue.

**Data curation:** Boxun Zhang, Yuan Chen.

**Formal analysis:** Maoyi Yang, Zhipeng Hu.

**Investigation:** Liangjun Yang, Boxun Zhang, Yuan Chen.

**Methodology:** Maoyi Yang, Zhipeng Hu, Rensong Yue.

**Project administration:** Rensong Yue

**Software:** Maoyi Yang, Zhipeng Hu.

**Visualization:** Liangjun Yang.

**Writing – original draft:** Maoyi Yang.

**Writing – review and editing:** Rensong Yue and Liangjun Yang.

Zhipeng Hu orcid: 0000-0003-1524-6452.
